# Leaders’ Expressed Humility and Followers’ Feedback Seeking: The Mediating Effects of Perceived Image Cost and Moderating Effects of Power Distance Orientation

**DOI:** 10.3389/fpsyg.2018.00563

**Published:** 2018-04-18

**Authors:** Jing Qian, Xiaoyan Li, Baihe Song, Bin Wang, Menghan Wang, Shumeng Chang, Yujiao Xiong

**Affiliations:** ^1^Department of Human Resource Management, Business School, Beijing Normal University, Beijing, China; ^2^School of Business, Jiangxi Normal University, Nanchang, China

**Keywords:** humility, perceived image cost, feedback seeking, power distance orientation

## Abstract

We developed and tested a model to identify the role of leaders’ expressed humility on employees’ feedback-seeking processes. The data used in our study was from a sample of 248 employees and 57 of their immediate supervisors. The results revealed that: (1) leader’s expressed humility positively related to employees’ feedback seeking mediated by employees’ perceived image cost; and (2) power distance orientation moderated the relationship between leader’s expressed humility and employees’ perceived image costs, such that the relationship was stronger when the power distance orientation was lower rather than higher. The results offer new insight into potential managerial practices that aim at stimulating feedback seeking. We conclude with a discussion for future research.

## Introduction

“The x-factor of great leadership is not personality; it’s humility.”

Jim Collins

Employees’ feedback-seeking behavior plays an important role in enhancing individual performance and creativity in the face of today’s dramatically changing business environment (Ashford et al., 2003; Anseel et al., 2015). Over the past three decades, scholars and practitioners have been looking for effective ways to promote employees’ feedback seeking. Among these researchers, many have focused on the role of leaders in the feedback-seeking process. This is because leaders have an important influence on employees’ work lives and they are often considered to be critical feedback sources (see the reviews by Ashford et al., 2003 and Anseel et al., 2015). Not surprisingly, empirical studies therefore have begun to identify the role of leadership behaviors in generating employees’ feedback seeking (e.g., VandeWalle et al., 2000; Chen et al., 2007; Qian et al., 2012). Recently, in research on leadership there has been an interest in leaders’ personal traits (e.g., humility), which are regarded as important factors in effective organizational leadership (Cameron et al., 2003; Owens et al., 2013). Humility has been viewed as a meta-virtue that can shape other virtues and influence leaders’ management style and their employees’ positive work outcomes (Argandona, 2015; Oc et al., 2015; Owens et al., 2015). It is not surprising that humility has attracted more and more attention from organizational researchers (Boje et al., 2004; Owens et al., 2013; Ou et al., 2015; Rego et al., 2018). Recent research emphasizes the interpersonal and behavioral influences on humility and suggests that humility is particularly critical to exchanges of information in supervisor-subordinate dyads (Owens et al., 2013). Nielsen et al. (2010) suggest that individuals with humility are actively engaged in taking advantage of information sought from interactions with others. Indeed, one important aspect of humility is manifested by showing openness to others’ ideas, suggestions, and desires of asking for help (Owens et al., 2013). Despite research on the theoretical significance of leaders’ humility and the potential encouragement of employees’ feedback seeking, to date, few studies have focused on understanding how leaders’ humility is associated with followers’ feedback-seeking behaviors (i.e., an important way to ask for information).

A leader’s expressed humility refers to individual characteristics displayed in interpersonal interactions that connote his or her willingness to acquire self-knowledge, appreciation of others’ strengths or contributions and teaching ability (Owens et al., 2013). We focus on employees’ seeking of feedback from supervisors and examine leaders’ expressed humility a potential antecedent of such behavior. We suggest that employees would be encouraged to seek feedback from supervisors if they perceive their leaders’ expressed humility. Humility is considered a key personal trait that can facilitate the development of other positive individual qualities (Oc et al., 2015; Rego et al., 2018) and hence can have significant effects on several important organizational outcomes (e.g., Morris et al., 2005; Owens and Hekman, 2012). Organizations are badly in need of leaders with greater humility as such leaders are better at managing today’s increasingly dynamic and complex organizational structures (Weick, 2001). Recently Owens et al. (2013) integrated the findings of previous studies and redefined humility as expressed humility by focusing on the observable interpersonal and behavioral aspects of humility. After they established a psychometrically robust measurement of it, empirically investigating the effects of leaders’ expressed humility in organizations has attracted great interest. For example, Ou et al. (2015) reported that CEOs’ humility positively relates to firm performance. Rego et al. (2018) found that leaders’ humility positively relates to team effectiveness.

In order to better understand how leaders’ expressed humility influences employees’ feedback seeking, we further examine employees’ perceived image costs as a mediator in this relationship. Employees’ perceived image costs refer to the potential costs incurred by asking for feedback, which may damage one’s image in front of others (Ashford, 1986). A cost-value framework has been used in most research on feedback seeking to interpret the underlying mechanisms of the feedback-seeking process (Anseel et al., 2015). In the present study, we focus on the role of perceived image costs in the feedback-seeking process. This is because the motivation to protect one’s image is one of the three fundamental motives of feedback seeking, which can directly influence individuals’ feedback behaviors (Ashford et al., 2003; Hays and Williams, 2011). More importantly, scholars argue that feedback source characteristics of leadership influences individuals’ feedback seeking behaviors through their effects on the costs related to feedback seeking (Anseel et al., 2015). Not surprisingly, then, perceived image cost has been examined as a mediator in explaining why and how leaders could encourage or discourage feedback seeking behavior (e.g., Choi et al., 2014; Chun et al., 2014). For example, the study by Chun et al. (2014) demonstrated that perceived cost mediates the relationship between leader-member exchange quality and employees’ feedback seeking. The study by Choi et al. (2014) found that perceived cost mediates the relationship between employees’ affect-based trust in their leaders and employees’ feedback seeking. Following this line of reasoning, we examine the mediating effect of perceived image cost in order to specify the process through which leaders’ expressed humility could generate employees’ feedback seeking.

**FIGURE 1:**
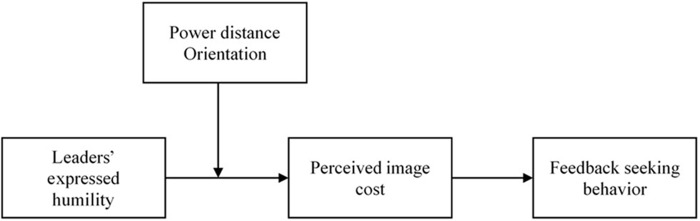
Theoretical model.

Additionally, we investigate power distance orientation as a moderator in the relationship between leaders’ expressed humility and employees’ perceived image cost. Power distance orientation refers to the extent to which individuals can accept the unequal distribution of power within organizations (Dorfman and Howell, 1988; Clugston et al., 2000). Power distance orientation is proposed as an individual cultural value that could have important influences on employees’ work experiences (Lian et al., 2012). Previous studies have identified the moderating impacts of power distance orientation in the work context (e.g., Zhang and Begley, 2011; Qian et al., 2012; Lee and Antonakis, 2014; Vidyarthi et al., 2014; Lin et al., in press). In the present study, we examine the moderating effect of power distance orientation on the relationship between leaders’ expressed humility and employees’ perceived image cost to advance this research line and to help find the boundary conditions for the effectiveness of leaders’ expressed humility on the feedback-seeking process. The hypothesized model is presented in **Figure 1**.

## Theory and Hypotheses

### Leaders’ Expressed Humility and Feedback Seeking

In the present study, we suggest that employees’ feedback seeking could benefit from leaders’ expressed humility. First, a leader’s humility expresses the leader’s desire to achieve accurate self-awareness (Owens et al., 2013). To develop such an awareness, leaders with high expressed humility may focus on interactions in organizations through which they could receive self-evaluative information. They interact with employees seriously and have the potential to build and maintain a higher-quality and more credible two-way feedback channel (Owens et al., 2013). As a result, employees would be motivated to seek feedback.

Second, a leader’s expressed humility contains a genuine appreciation of others’ strengths or abilities (Owens et al., 2013; Rego et al., 2018). Leaders who possess high expressed humility tend to give credit to employees’ extra efforts (Owens et al., 2013). They are more likely to notice and encourage the positive behaviors and initiative of their subordinates. Working with such leaders, employees could perceive that their efforts are expected and valued and may consider their leaders as secure and reliable sources for feedback.

Third, leaders with high expressed humility are considered to have high teachability (Owens et al., 2013). They have a strong willingness to learn from others so that they are more open and receptive to their employees’ ideas, advice, or information (Rego et al., 2018). For humble leaders, employees’ feedback-seeking behavior is more likely to be seen as a beneficial practice that may contribute to learning and development for both supervisors and subordinates. As such, humble leaders could create a supportive environment for personal learning and development (Sousa and van Dierendonck, 2017), which could encourage employees to engage in feedback seeking. Accordingly,

Hypothesis 1: Leaders’ expressed humility positively relates to employees’ feedback seeking.

### Mediating Effect of Perceived Image Cost

By decreasing employees’ perceived image cost, humble leaders could promote feedback seeking by employees. First, employees may view a humble leader as a role model. Previous scholars have stressed the notion that a leader’s expressed humility positively relates to collective humility in organizations (Owens and Hekman, 2016). In the present study, we argue that employees may imitate their leader’s humility behaviors (Owens and Hekman, 2016). Specifically, when humble leaders show their determination to achieve accurate self-awareness, employees may also be encouraged to pursue their own self-awareness (Qian et al., 2012). In line with this, employees would be more likely to consider the potential value of feedback seeking in terms of achieving accurate self-awareness and be less likely to take its possible cost of image damage into account, thus feeling motivated to seek feedback.

Second, humble leaders believe that everybody has his/her weaknesses and they often show great tolerance for them (Morris et al., 2005; Sousa and van Dierendonck, 2017). In fact, humble leaders even have the courage to disclose their own limitations or weaknesses (Owens et al., 2013). Being inspired by humble leaders, employees may be more accepting and less concerned about the exposure of their weaknesses. This could foster more transparent two-way communications and develop higher-quality interpersonal interactions between leaders and followers. Consequently, employees would be more willing to ask for feedback from their leaders.

Third, the high teachability of humble leaders could send signals to employees that they encourage learning behaviors in the work context (Owens et al., 2013). Previous research suggest that being teachable may help make learning behaviors normal and guaranteed in the workplace (Owens and Hekman, 2012). Feedback-seeking behavior is one of the essential learning behaviors that are normally tied to mistakes and risks. Employees under the supervision of such teachable leaders are more likely to perceive this learning behavior as less costly in terms of damaging one’s image. As they perceive less image cost, employees are more willing to learn from humble leaders by seeking feedback. Accordingly,

Hypothesis 2: Employees’ perceived image cost mediates the positive relationship between leaders’ expressed humility and employees’ feedback seeking.

#### Moderating Effect of Power Distance Orientation

Previous studies have pointed out that power distance orientation could directly impact how individuals interact with others (e.g., Hofstede, 2001; van der Vegt et al., 2005). The employees with low power distance orientation tend to interact with high-status members more equally (Hofstede, 2001). This could help shorten the psychological distance between leaders and followers (Yuan and Zhou, 2015). As such, employees with low power distance orientation are more willing to be influenced and assimilated by humble leaders. More specifically, leaders’ humble behaviors may gain better recognition and acceptance from these employees. The low power distance orientation employees are more likely to have a greater understanding of humble leaders’ values and may internalize them as their own values. As a result, they are strongly and increasingly affected by humble leaders. They perceive humble leaders as more approachable and feel comfortable to interact equally with them, generating an increased sense of security (Zhang and Begley, 2011). The reduction of employees’ perceived image cost generated by a leader’s expressed humility, therefore, would be more significant.

In contrast, employees who have high levels of power distance orientation are more likely to have an expectation that their supervisors should behave as autocratic and powerful leaders (e.g., Hofstede, 2001; House et al., 2004; Yuan and Zhou, 2015). However, expressed humility is, by nature, a pattern of behaviors expressing low power distance orientation (Yuan and Zhou, 2015). Humble leaders most frequently maintain a low profile in front of followers (Ou et al., 2014). This does not live up to high power distance orientation employees’ expectations (Huang et al., 2011). As a result, leaders’ humble behaviors are more likely to face resistance from high power distance orientation employees (Yuan and Zhou, 2015). As such, high power distance orientation employees may be weakly influenced by humble leaders; thus, the effect of a leader’s expressed humility on high power distance orientation employees’ perceived image cost would be less effective. Additionally, employees with high power distance orientation seem to take their leaders too seriously; thus, they cannot find interpersonal interactions with the leaders to be easy (Qian et al., 2012). Even though humble leaders show sufficient tolerance toward individuals’ limitations and weaknesses, employees possessing high power distance orientation may still consider the exposure of their imperfect image in front of leaders to be unacceptable. Therefore, high power distance orientation would mitigate the positive influence of leaders’ expressed humility on reducing employees’ perceived image cost. Accordingly,

Hypothesis 3: The negative relationship between leaders’ expressed humility and employees’ perceived image cost will be moderated by power distance orientation in such a way that the relationship will be stronger when power distance orientation is lower rather than higher.

## Materials and Methods

### Participants and Procedure

The data used in this study were collected from a hotel group situated in a large city in China. One of the authors was invited to lead a training program for this hotel group. The participants were 64 supervisors who had attended the training program and they were recruited at the end of the training program. With the support of the human resources department we randomly selected five subordinates of each supervisor from a list of names. Separate questionnaires were prepared for and distributed to 64 supervisors and their 320 subordinates; all were completed by paper and pencil. Supervisor participants and subordinate participants finished supervisor questionnaires and subordinate questionnaires respectively. Supervisor participants were asked to rate their perceptions of the subordinates’ feedback seeking behavior, while subordinate participants were asked to rate their perceptions of the immediate supervisor’s humility, perceived image cost of feedback seeking, and power distance. Demographic information was collected from both groups.

Participants completed the survey voluntarily. After completing the survey, each participant was given a small gift (a pen costing 15 RMB) as an incentive. We ensured participants’ confidentiality by providing a return envelope with seal tape for participants to seal the finished questionnaire; participants were instructed to complete the questionnaires, enclose them in the sealed envelopes, and return them at a company-wide meeting which was held 2 weeks later for all employees (both supervisory and non-supervisory). Subordinates’ responses were matched with their immediate supervisors’ responses by numerical codes and not names. We obtained participants’ written informed consent before the implementation of data collection. All these procedures were conducted in accordance with the ethical standards of the institutional and/or national research committee and with the 1964 Helsinki declaration and its later amendments or comparable ethical standards with written informed consent from all subjects. The present study was approved by the Human Research Ethics Committee (HREC) at the Business School of Beijing Normal University.

Of all those surveyed, 57 supervisors and 248 subordinates returned questionnaires which were used for hypothesis testing (i.e., 89.06 and 77.5% response rate, respectively). Among the final sample of subordinate respondents, 62.1% were men. The average age, organizational tenure, and team tenure were 32.58 (*SD* = 8.28), 6.31 (*SD* = 3.99), and 4.18 (*SD* = 2.21) years, respectively.

### Measures

The measures used in the present study were originally constructed in English. In order to ensure equivalence of the measures in the Chinese and the English versions of the survey instrument, we performed a standard translation and back-translation procedure, following Brislin (1980). The Chinese version was subsequently pilot-tested on employees of the participating organization who were excluded from the final sample.

#### Leaders’ Expressed Humility

We measured leaders’ expressed humility by using the nine-item expressed humility scale developed by Owens et al. (2013). Response options ranged from 1 “strongly disagree” to 7 “strongly agree.” A sample item is, “This leader shows a willingness to learn from others” (Coefficient alpha = 0.93).

#### Perceived Image Cost

We measured employees’ perceived image cost by using Ashford’s (1986) nine-item perceived image cost scale. Response options ranged from 1 “strongly disagree” to 7 “strongly agree.” A sample item is, “I would not be nervous about asking my boss how he/she evaluates my behaviors (R)” (Coefficient alpha = 0.78).

#### Feedback-Seeking Behavior

We measured employees’ feedback seeking by using the five-item feedback-seeking behavior scale developed by VandeWalle et al. (2000). Response options ranged from 1 “never” to 7 “always.” A sample item is, “How often does this subordinate ask you for feedback about his or her overall job performance?” (Coefficient alpha = 0.79).

#### Power Distance Orientation

We measured power distance orientation by using Dorfman and Howell’s (1988) five-item power distance orientation scale. Response options ranged from 1 “strongly disagree” to 5 “strongly agree.” A sample item is, “It is frequently necessary for a manager to use authority and power when dealing with subordinates” (Coefficient alpha = 0.86).

#### Control Variables

We controlled for participants’ age, gender, education level, and company tenure for several reasons. First, previous studies have demonstrated that certain demographic differences, such as age, gender, and organizational tenure, could exert influences on individuals’ feedback-seeking behavior (Barner-Rasmussen, 2003; Finkelstein et al., 2003; Miller and Karakowsky, 2005). Second, in accordance with previous studies of humility (Ou et al., 2014; Owens et al., 2015; Owens and Hekman, 2016; Rego et al., 2018), we included these four control variables (i.e., participants’ age, gender, education level, and company tenure) when testing the hypotheses. Age and company tenure were measured by number of years. Gender was coded 0 for “female” and 1 for “male.” As education level is categorical variable, we created three dummy variables. Dummy1 was coded as 1 = high school, 0 = others; Dummy 2 was coded as 1 = bachelor, 0 = others; Dummy 3 was coded as 1 = master, 0 = others.

### Analytic Strategy

First, we carried out confirmatory factor analysis in AMOS 22.0 to assess the distinctiveness of the variables studied. As employees were nested in teams, we examined the ICC1 and ICC2 to determine whether multilevel analysis was appropriate. We then conducted regression analyses in SPSS 22 to test our model; the results are reported in **Table 3**. We also examined whether the indirect association between humility and feedback seeking using the PROCESS macro (Hayes, 2013) to implement moderated mediation analysis.

## Results

### Validity of the Scales

**Table 1** presents the confirmatory factor analysis results. Given the relatively small sample size, relative to the number of parameters, the use of parcels is appropriate (Matsunaga, 2008). We used factorial algorithm (averageing the highest and lowest loadings to establish the first indicator, Matsunaga, 2008) to create parcels for each latent variables, and finally all the scales were trimmed to three parcels. As shown, the four-factor model fitted the data well (χ^2^ = 108.01; df = 48; χ^2^/df = 2.25; RMSEA = 0.07; CFI = 0.97; TLI = 0.96). We compared the fit of the hypothesized four-factor model with that of a null model, two three-factor models, one two-factor model and one one-factor model. As shown in **Table 1**, the four-factor model fitted the data better than the other models, providing evidence of the distinctiveness of the constructs of leaders’ expressed humility, and employees’ perceived image cost, power distance orientation and feedback-seeking tendency.

**Table 1:** Results of confirmatory factor analyses.

Model	Factor	χ^2^	df	χ^2^/df	Δχ^2^	CFI	TLI	RMSEA
Four-factor model		108.01^∗∗∗^	48	2.25	_	0.97	0.96	0.07
Three-factor model	Humility and perceived image cost were combined into one factor	256.00^∗∗∗^	51	5.02	147.30^∗∗∗^	0.90	0.87	0.13
Three-factor model	Humility and power distance were combined into one factor	1022.41^∗∗∗^	51	20.05	914.41^∗∗∗^	0.53	0.39	0.28
Two-factor model	Humility, power distance, and perceived image cost were combined into one factor	1167.54^∗∗∗^	53	22.03	1059.53^∗∗∗^	0.46	0.33	0.29
One-factor model	Humility, power distance, perceived image cost, and feedback seeking behavior were combined into one factor	1360.39^∗∗∗^	54	25.19	1252.38^∗∗∗^	0.37	0.23	0.31


*CFI, comparative fit index; TLI, Tucker-Lewis coefficient; NNFI, non-normed fit index; RMSEA, root mean square error of approximation. ^∗∗∗^*p* < 0.001.*

### Hypothesis Testing

#### Descriptive Statistics

The means, standard deviations, reliabilities, and correlations for the study variables are presented in **Table 2**. Leaders’ expressed humility was positively related to employees’ feedback-seeking (*r* = 0.13, *p* < 0.05) and negatively related to employees’ perceived image cost (*r* = -0.20, *p* < 0.001); employees’ perceived image cost was negatively related to employees’ feedback-seeking behavior (*r* = -0.15, *p* < 0.05).

**Table 2:** Means, standard deviations, reliabilities, and correlations among study variables.

	*M*	*SD*	1	2	3	
(1) Leaders’ expressed humility	5.78	0.66	(0.93)			
(2) Perceived image cost	2.20	0.58	-0.20^∗∗∗^	(0.71)		
(3) Feedback seeking	4.21	0.52	0.13^∗^	-0.15^∗^	(0.79)	
(4) Power distance orientation	3.13	0.87	-0.05	-0.02	-0.07	(0.82)


**N* = 248. Cronbach’s alpha reliabilities are in parentheses on the diagonal. ^∗^*p* < 0.05; ^∗∗^*p* < 0.01; ^∗∗∗^*p* < 0.001.*

#### Mediating Effect Tests

Employees are nested in teams, so we estimated our models using a multilevel method. First, we tested the between-group and within-group variance in the outcome (i.e., feedback-seeking behavior). Our results showed that ICC1 was 0.065 and ICC2 was 0.25, *p* > 0.05, indicating that analysis of within-group correlations was more appropriate, so like previous studies (van der Vegt et al., 2003; Lam et al., 2007), we have reported single-level regression results.

As shown in **Table 3**, first, taking employees’ perceived image cost as the independent variable, leaders’ expressed humility was negatively related to employees’ perceived image cost (*b* = -0.25, *p* < 0.001; model 2). Taking employees’ feedback seeking behavior as the independent variable, when we controlled for those demographic variables (i.e., age, gender, education level, and tenure), leaders’ expressed humility positively predicted employees’ feedback seeking behavior (*b* = 0.09, *p* < 0.1; model 5), providing support for Hypothesis 1. When we entered the mediator into the model, employees’ perceived image cost was negatively related to employees’ feedback seeking behavior (*b* = -0.12, *p* < 0.05; model 6). However, in model 6, the coefficient of leaders’ expressed humility became insignificant, indicating that the perceived image cost fully mediated the relationship between leaders’ expressed humility and employees’ feedback seeking behavior.

**Table 3:** Results of regression analysis.

	Perceived image cost			Feedback seeking behavior		
		
	Model 1	Model 2	Model 3	Model4	Model 5	Model 6
Gender	0.04	0.02	0.03	-0.15*	-0.14	-0.14
Age	-0.00	-0.00	-0.00	-0.00	-0.00	-0.00
Dummy 1	-0.05	-0.01	0.02	-0.56	-0.56	-0.57
Dummy 2	-0.02	0.04	0.04	-0.56	-0.57	-0.57
Dummy 3	-0.24	-0.17	-0.12	-0.41	-0.42	-0.44
Tenure	-0.02	-0.02	-0.02	0.00	0.00	0.00
Leaders’ expressed humility		-0.25***	-0.17***		0.09^†^	0.07
Power distance		-0.05	-0.04			
Leaders’ expressed humility × power distance			0.06^†^			
Perceived image cost						-0.10^†^
*R*^2^	0.04	0.12	0.13	0.04	0.05	0.06
Δ *R*^2^	0.04	0.08***	0.02^†^	0.04	0.02^†^	0.01^†^


**N* = 248; ^†^*p* < 0.1; ^∗^*p* < 0.05; ^∗∗^*p* < 0.01; ^∗∗∗^*p* < 0.001. We create three dummy variables for education level: Dummy1 was coded as 1 = high school, 0 = others; Dummy 2 was coded as 1 = bachelor, 0 = others; Dummy 3 was coded as 1 = master, 0 = others.*

#### Moderating Effect Tests

To test the moderating influence of power distance orientation, we entered variables into regression analysis at three steps: (1) the control variables (i.e., gender, age, educational level, and tenure); (2) employees’ perceived image cost, the moderator (i.e., power distance orientation); and (3) the two-way interactive term (i.e., leaders’ expressed humility × power distance orientation). As shown in **Table 3**, the two-way interactive term (i.e., leaders’ expressed humility × power distance orientation) was positively related to employees’ perceived image cost (*b* = 0.07, *p* < 0.1; model 3). Thus, Hypothesis 3 (i.e., the moderating effect) was supported.

**FIGURE 2:**
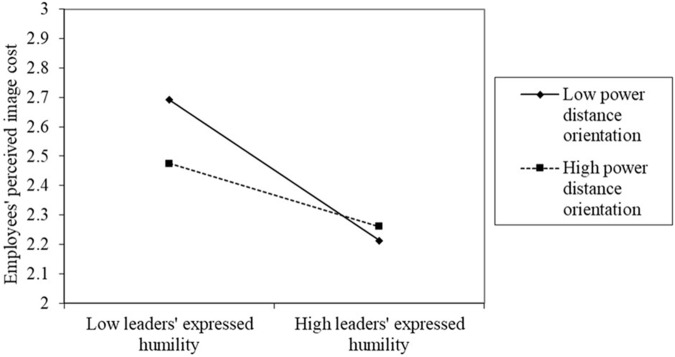
The moderating effect of power distance orientation.

When interpreting the specific moderating influence of power distance orientation, we calculated regression equations for three steps: first, we followed Aiken and West’s (1991) method to standardize the data; second, we defined high power distance orientation as plus one standard deviation from the mean and defined low power distance orientation as minus one standard deviation from the mean, based on Cohen and Cohen’s (1983) research; finally, the regression equations were calculated for the relationship between leaders’ expressed humility and employees’ perceived image cost for high and low levels of power distance orientation. As shown in **Figure 2**, the linear relationship between a leader’s expressed humility and employees’ perceived image cost was stronger for employees possessing low levels of power distance orientation and weaker for employees possessing high levels of power distance orientation. Thus, Hypothesis 3 was fully supported.

#### Moderated Mediation

We also conducted moderated mediation analysis using used PROCESS macro developed by Hayes (2013) to investigate whether power distance orientation moderated the indirect effect. As shown in **Table 4**, power distance orientation failed moderating the link between leaders’ expressed humility and employees’ feedback-seeking behavior.

**Table 4:** Bootstrap estimates of indirect effect at -1 SD, the mean, and the +1 SD levels of moderator.

Moderator	Indirect effect (b, Boot SE)	95% CI (lower–upper level CI)
Power distance		
Leaders’ expressed humility – perceived image cost – feedback seeking behavior		
-1 SD	0.024 (0.014)	-0.0010 to 0.0545
Mean	0.018 (0.010)	-0.0004 to 0.0403
+1 SD	0.012 (0.008)	0.0007 to 0.0353


#### Supplementary Analysis

As two main paths are “marginally significant” (i.e., the coefficient of the interactive term and the relationship between perceived image cost and feedback seeking behavior), we conducted the supplementary analysis without control variables to detect the effects more powerfully. As shown in **Table 5**, the interactive term of leaders’ expressed humility × power distance orientation was still “marginally significant” (*b* = 0.06, *p* = 0.07; model 8), and the relationship between perceived image cost and feedback seeking behavior became significant (*b* = -0.12, *p* < 0.05; model 10). Following Cohen (1992), we also reported the effect size in **Table 5**.

**Table 5:** Supplementary analysis.

	Perceived image cost		Feedback seeking behavior	
		
	Model 7	Model 8	Model 9	Model 10
Leaders’ expressed humility	-0.157***	-0.17***	0.06^†^	0.04
Power distance	-0.02	-0.02	-0.04	-0.04
Leaders’ expressed humility × Power distance		0.06^†^	0.03	0.03
Perceived image cost				-0.12*
*R^2^*	0.07	0.09	0.02	0.04
Δ *R^2^*	0.07***	0.02^†^	0.02	0.02*
*f^2^*		0.02		0.02


**N* = 248; ^†^*p* < 0.1; ^∗^*p* < 0.05; ^∗∗^*p* < 0.01; ^∗∗∗^*p* < 0.001; f^2^ = Δ R^2^/(1-R^2^).*

## Discussion

In the present study, we developed and tested a model aiming to explicitly delineate the relationship between expressed humility and feedback-seeking behavior by exploring the underlying mediating mechanism as well as the boundary condition. The hypotheses in the present study were supported by the results, which revealed that: (1) leaders’ expressed humility positively relates to employees’ feedback seeking; (2) employees’ perceived image cost mediates the positive relationship between leaders’ expressed humility and employees’ feedback seeking; and (3) power distance orientation moderates the relationship between leaders’ expressed humility and employees’ perceived image cost, such that the relationship will be stronger when power distance orientation is lower rather than higher.

### Theoretical Implications

This study has several important theoretical implications. First, we have shown that humble leaders play a dynamic role in the feedback-seeking process by analyzing leaders’ expressed humility as an antecedent of employees’ seeking of feedback. Many studies have investigated relationships between leadership style and feedback seeking (e.g., VandeWalle et al., 2000; Chen et al., 2007; Qian et al., 2012), but leaders’ expressed humility has been surprisingly absent from consideration although humility is regarded as “a fundamental quality of a good manager and good management” (Argandona, 2015, p. 63). This may be because traditional studies of humility focus mainly on its intrapersonal aspects and consider humble leaders as passive and as having low self-esteem (Rego et al., 2018) and thus be less favored sources for employees seeking feedback. Recently, scholars have begun to investigate the interpersonal benefits of humility and suggest that it is particularly critical to exchanges of information in supervisor-subordinate dyads (Owens et al., 2013). Although scholars emphasize the importance of humility in information exchange between leaders and followers, some important issues remain unknown. Owens et al. (2013) redefined humility as expressed humility, which focuses on the interpersonal and behavioral aspects, and following empirical studies have examined the relationship between leaders’ expressed humility and workplace outcomes (e.g., Ou et al., 2015; Rego et al., 2018). Considering these recent studies (Owens et al., 2013; Ou et al., 2015; Rego et al., 2018), our modeling supports the idea that there are strong theoretical reasons to expect an association between humility and feedback-seeking behavior. This study represents the first attempt to examine empirically the relationship between expressed humility and feedback seeking. It thus extends knowledge of the antecedents of feedback-seeking behavior and contributes to research on unlocking the benefits of humility in organizations.

Second, employees’ perceived image cost has been considered and explored as a primary determinant of employees’ feedback seeking in a large number of previous research studies (e.g., Ashford, 1986; VandeWalle et al., 2000; Ashford et al., 2003; Qian et al., 2012; Choi et al., 2014; Chun et al., 2014; Anseel et al., 2015). We extend this topic by examining the mediating effect of employees’ perceived image cost on the humility-feedback seeking relationship. By doing so, the present study also identified the process through which humble leaders could influence employees’ feedback seeking. Third, we examined the contingency side of the humility-perceived image cost relation by addressing the exploratory question of whether individuals’ power distance orientation plays a moderating role. Previous scholars focused mainly on examining humility in Western cultures and have made considerable progress, while the influences and the contingency sides of humility in non-Western contexts are still a largely unknown area (Oc et al., 2015). To fill this gap, the present study examined the unique moderating effect of power distance orientation in a Chinese setting, suggesting that the positive influence of leaders’ expressed humility on reducing employees’ perceived image cost will be weaker when employees possess higher power distance orientation.

### Practical Implications

The present study also provides some important suggestions for managerial practice. First, our findings suggest that leaders’ expressed humility can effectively generate employees’ feedback seeking. Accordingly, organizations should attach importance to humility when selecting and training supervisors. With regard to selecting managers, organizations are suggested to take humility as an important criterion (Ou et al., 2015). In terms of how to develop humility among leaders, previous researchers have advised organizations to use systematic training programs (Ou et al., 2015). More specifically, some researchers suggest that personal humility may be potentially developed via giving reality-based feedback about one’s merits and demerits, or not overemphasizing one’s performance and contributions in his/her last job (Exline and Geyer, 2004; Owens et al., 2013). Second, our findings also suggest that employees’ perceived image cost could mediate the humility-feedback seeking relation. Thus, besides learning to behave as humble leaders, supervisors should also make the extra effort to master how to alleviate employees’ concerns about damage to their image. To achieve this, supervisors can behave professionally, listen to their employees’ words empathetically, and counsel their subordinates sincerely (Choi et al., 2014). Third, our findings concerning the moderating influence of power distance orientation suggest that supervisors should pay particular attention to individuals’ different cultural values when trying to exert influence. It is suggested that supervisors act differently on the basis of the employee’s special cultural values (Lin et al., in press). For example, supervisors may show their powerful aspects to employees with high power distance orientation in order to meet their cultural expectations (Yuan and Zhou, 2015) and show genuine concern about their daily lives in order to relieve their tension in front of leaders (Lin et al., in press).

### Limitations

The present study also has several limitations. First, our data was solely collected from one Chinese company. This may limit the general applicability of our findings. For example, China is considered to have high power distance cultural contexts (e.g., Yuan and Zhou, 2015), which may influence individuals’ cultural values. Thus, it may be speculated as to whether our findings concerning the moderating role of power distance orientation are applicable to other cultures. In the present study, the average rating (using a seven-point Likert scale) of perceived image cost was lower (*M* = 2.20, *SD* = 0.58) than that of previous studies (Choi et al., 2014; Chun et al., 2014). This might attribute to the characteristics of our sample. Employees working in hotels play an important role in service encounters. In other to deliver high quality service, they often obtain various forms of feedback information about themselves. The perceived image cost of seeking feedback is thus lower in this workplace. We encourage future researches to collect data from other types of organizations in diversified industries and/or cultures.

Second, although our hypotheses are supported, we cannot draw definitive conclusions. As two paths are “marginally significant,” we conducted supplementary analysis to examine our model without control variables and computed the effect sizes. However, the small effect sizes may contribute to Type II error (Cohen, 1992). Thus, the current need larger sample sizes to provide robust results. Besides, because we apply cross-sectional design in the present study, the causal inferences of the positive relationship between leaders’ expressed humility and employees’ feedback seeking cannot be determined. Supervisors may behave more humbly in front of subordinates who seek feedback from them more proactively and frequently. Additional quasi-experimental or longitudinal research is needed to clarify this issue. Third, the present study focuses on the cost-minimizing role of leaders’ expressed humility in the feedback-seeking process. Given the important roles of the cost-value framework in interpreting the underlying mechanisms of the feedback-seeking process (Anseel et al., 2015), researchers may also take the potential mediating effect of value perceptions into consideration. Indeed, many previous effective leadership studies simultaneously investigated perceived cost and value as mediators in their theoretical models (Teunissen et al., 2009; Qian et al., 2012). Qian et al. (2012), for example, argued that authentic leaders stimulate followers’ feedback seeking from supervisors via decreasing employees’ perceived image costs as well as increasing the perceived value. Therefore, we encourage future studies to measure the perceived value of feedback seeking and examine its interaction effect with the perceived cost of feedback seeking to extend the present model.

Third, in this study we only controlled for the effects of variance in participants’ age, gender, educational level and company tenure, but humility is seen as an important feature of certain effective leadership styles (e.g., authentic leadership and transformational leadership) that have been shown have a positive influence on feedback-seeking behavior (Qian et al., 2012; Anseel et al., 2015). In future, therefore, researchers may wish to control for variance in these effective leadership styles in order to identify the unique contribution of humility to feedback-seeking as this would provide a more rigorous test of our model.

Finally, in the present study, we only used one other-report approach (i.e., subordinate-report approach) to measure leader’s expressed humility. Although previous studies argue that other-report measures provide more valid assessments than self-report measures (Rego et al., 2018), the present study would be better elaborated by including other types of raters. For example, future studies may measure leader’s expressed humility by using self-reported, subordinate-reported, and peer-reported approaches (Rego et al., 2018). By doing so, scholars may identify the differences between informant-rated humility and self-reported humility (e.g., Rego et al., 2018). In addition, future studies may also measure leader’s expressed humility by using a consensus assessment among other-report ratings and self-report ratings (Davis et al., 2010; Meagher et al., 2015), extending the present study to a multi-level model.

## Conclusion

Identifying the dynamic role of leadership in generating employee feedback seeking has increasingly attracted attention from scholars. Our findings advance this rising research line by suggesting that leaders’ expressed humility could generate employee feedback seeking by decreasing employees’ perceived image cost. Our findings also suggest power distance orientation as one of the important boundary conditions for the effectiveness of leaders’ expressed humility on the feedback-seeking process.

## Ethics Statement

All procedures performed in studies involving human participants were in accordance with the ethical standards of the institutional and/or national research committee and with the 1964 Helsinki declaration and its later amendments or comparable ethical standards with written informed consent from all subjects. This research was approved by the Human Research Ethics Committee (HREC) at Business School, Beijing Normal University.

## Author Contributions

JQ and BS substantially contributed to the conception, the design of the work, and the preparation of the draft. XL, MW, SC, and YX reviewed it critically and gave important intellectual input. BW contributed to the analysis and interpretation of the data.

## Conflict of Interest Statement

The authors declare that the research was conducted in the absence of any commercial or financial relationships that could be construed as a potential conflict of interest.
